# Eating Frequency, Food Intake, and Weight: A Systematic Review of Human and Animal Experimental Studies

**DOI:** 10.3389/fnut.2015.00038

**Published:** 2015-12-18

**Authors:** Hollie A. Raynor, Matthew R. Goff, Seletha A. Poole, Guoxun Chen

**Affiliations:** ^1^Department of Nutrition, University of Tennessee, Knoxville, TN, USA; ^2^Nestlé Health Science, Florham Park, NJ, USA

**Keywords:** eating frequency, grazing, food intake, body weight, appetite, human and animal models

## Abstract

Eating frequently during the day, or “grazing,” has been proposed to assist with managing food intake and weight. This systematic review assessed the effect of greater eating frequency (EF) on intake and anthropometrics in human and animal experimental studies. Studies were identified through the PubMed electronic database. To be included, studies needed to be conducted in controlled settings or use methods that carefully monitored food intake, and measure food intake or anthropometrics. Studies using human or animal models of disease states (i.e., conditions influencing glucose or lipid metabolism), aside from being overweight or obese, were not included. The 25 reviewed studies (15 human and 10 animal studies) contained varying study designs, EF manipulations (1–24 eating occasions per day), lengths of experimentation (230 min to 28 weeks), and sample sizes (3–56 participants/animals per condition). Studies were organized into four categories for reporting results: (1) human studies conducted in laboratory/metabolic ward settings; (2) human studies conducted in field settings; (3) animal studies with experimental periods <1 month; and (4) animal studies with experimental periods >1 month. Out of the 13 studies reporting on consumption, 8 (61.5%) found no significant effect of EF. Seventeen studies reported on anthropometrics, with 11 studies (64.7%) finding no significant effect of EF. Future, adequately powered, studies should examine if other factors (i.e., disease states, physical activity, energy balance and weight status, long-term increased EF) influence the relationship between increased EF and intake and/or anthropometrics.

## Introduction

Approximately two out of every three adults in the U.S. are overweight or obese (1). The high prevalence of overweight and obesity negatively affects the health of the population, as obese individuals are at increased risk for developing several chronic diseases, such as type 2 diabetes (2), cardiovascular disease, and certain forms of cancer (3–5). Due to its impact on health, medical costs, and longevity, obesity is considered to be the number one health problem in the U.S. (6), and has become a public health priority (7).

One key area in obesity treatment is reducing energy intake (4). Ideally, the dietary prescription provided for reducing energy intake aids with appetite control, thereby enhancing ability to consume less energy, producing greater weight loss, and improving long-term weight loss maintenance. One dietary strategy that has long been proposed in the lay literature to improve appetite control and assist with weight management is increased eating frequency (EF) (i.e., eat small amounts of food every 2–3 h – “grazing”) (8, 9). However, while “grazing” is often suggested as a helpful strategy for managing hunger, the Dietary Guidelines Committee of 2010 stated that there is a lack of research in the area on EF and body weight and obesity, thus conclusions regarding an optimum EF prescription for weight management cannot be made and research on this topic is greatly needed (10). Therefore, within the scientific community there is agreement that the relationship between EF and management of food intake and weight is not clear (10).

The relationship between EF and body weight in humans was first examined with a cross-sectional investigation that was published in 1964 (11). In this study, an inverse relationship between self-reported EF and adiposity was found in 379 men aged 60–64 years (11). This study and other observational studies that soon followed led to the development of a hypothesis regarding EF and appetite control (12, 13). This hypothesis proposes that eating frequently during the day moderates variability in hunger sensations, potentially via reducing variability in hormonal response, making it easier to control energy intake and body weight (8, 9).

In the 50 years since the relationship of EF and weight status was initially investigated in humans, numerous observational studies have been conducted examining this relationship in adults and outcomes are very mixed [for review, see Bellisle and colleagues (12), McCrory and colleagues (13)]. One factor that has been suggested to be contributing to the mixed findings in observational studies is reporting bias that commonly occurs with self-reported dietary assessment (13). Under-reporting of energy intake is more common in individuals who are overweight or obese, and the number of eating occasions reported decreases as the magnitude of under-reporting increases (12). Thus, in observational studies, the relationship between EF and weight status may be an artifact of greater under-reporting of intake in those of a higher weight status (13). Due to this issue, to better understand the relationship between EF and weight status, it has been recommended that research on EF should move to experimental, rather than observational, design, so that the efficacy of different EFs on intake and weight status, particularly in regards to weight management, can be examined (9, 13).

While previous reviews have been published in the area of EF, intake, and/or weight, these reviews have included observational studies and/or failed to include animal experimental research (9, 12–18). Thus, to provide a comprehensive overview of experimental research that has been conducted in the area of greater EF, the purpose of this systematic review was to assess the effect of EF on intake and anthropometrics in human and animal experimental studies. Additionally, if measures of potential mechanisms by which greater EF may influence weight status (i.e., energy expenditure, appetite, and cardiometabolic/hormonal measures) were collected, results on these potential mechanisms are also reported.

## Methods and Materials

### Identification of Studies and Eligibility Criteria

Studies were identified by searching the PubMed electronic database from January to March 2014, and in November 2015. The search terms included were eating frequency, snacking, and feeding frequency AND appetite, satiety, satiation, energy intake, body weight, obesity, and metabolism. These terms were used as they are meaningful terms for both human and animal research, and thus could be used for both searches (human and animal). No constraints were set on date of publication or type of study; however, only articles published in English were reviewed. Identified abstracts were screened for duplicates. Abstracts identified were reviewed (Guoxun Chen, Matthew R. Goff, and Hollie A. Raynor) and full articles for abstracts meeting criteria were retrieved and further evaluated (Matthew R. Goff and Hollie A. Raynor). Discrepancies in decisions were discussed and resolved with Guoxun Chen and Seletha A. Poole. Additionally, bibliographies of identified reviewed studies (9, 12–15, 19) were examined to identify additional articles not in electronic databases.

### Inclusion Criteria for Studies

For human studies, only original research studies reporting the influence of a manipulation of increasing EF on a measure of energy intake, as assessed via daily energy consumed or energy intake during an *ad libitum* meal, or an anthropometric outcome, as assessed by body mass index (BMI) or body weight, in adults (sample only included those aged ≥18 years) were considered for inclusion. All experimental designs (i.e., where the independent variable of EF was manipulated in the investigation) were eligible for inclusion. Studies that only included participants with a health condition (i.e., type 2 diabetes, hypercholesterolemia, etc.), aside from being overweight or obese, were not included in this review. Due to the issue of under-reporting with self-reported dietary data, studies with only self-reported dietary data reporting on EF or energy intake related to the experimental manipulation were removed. Thus, the review only included studies that were conducted in a laboratory setting or in which participants’ food intake was carefully monitored (i.e., food was packaged and provided to participants in appropriate portions to consume).

Inclusion criteria for animal studies were similar to that of human studies, in that only original research studies reporting the influence of a manipulation of increasing EF on a measure of food intake or body weight were considered for inclusion. All experimental designs were eligible for inclusion. Studies that only included animal models representing glucose or lipid metabolism disease states were not included in this review. There was no restriction on length of feeding manipulation to be included in the review.

### Data Extraction

For the human studies, general study characteristics (author, year of publication, study design, and length of study), characteristics of study population (age, gender, BMI), experimental manipulation (EF and diet prescription), study outcomes (energy intake and anthropometrics), and potential mechanisms (energy expenditure, self-reported appetite, and cardiometabolic/hormonal measures) were extracted from included studies. Hollie A. Raynor and Seletha A. Poole independently extracted data from each study. Discrepancies regarding data extraction were resolved by discussion.

For the animal studies, general study characteristics (author, year of publication, comparison groups, and testing duration), characteristics of study population (species, strain), experimental manipulation (EF and diet prescription), study outcomes (food intake and anthropometrics), and potential mechanisms (cardiometabolic/hormonal measures) were extracted from the included studies. Guoxun Chen and Matthew R. Goff independently extracted data from each study. Discrepancies regarding data extraction in both human and animal studies were resolved by discussion.

### Outcomes of Interest

For human studies, extracted studies were divided into two categories based on the setting of the study: research or field. All identified studies reported on at least one of the primary outcomes (energy intake, anthropometrics). Energy expenditure (dietary-induced thermogenesis or total energy expenditure), self-reported appetite (hunger, fullness, and satiety), and/or cardiometabolic/hormonal measures (glucose, insulin, cholesterol, high-density lipoprotein, low-density lipoprotein, triglyceride, triacylglycerol, gastric inhibitory polypeptide, ghrelin, leptin, glucagon-like peptide-1, peptide YY, and free fatty acids) were reported as potential mechanisms or indicators of potential mechanisms in the relationship between EF, energy intake, and anthropometrics.

For animal studies, extracted studies were divided into two categories based on testing duration: EF studies conducted <1 month and EF studies conducted >1 month (month was defined as a single 30-day period). All identified studies reported at least one of the previously mentioned outcomes (food intake or body weight). Potential mechanisms regarding the relationship between EF and intake or weight, such as cardiometabolic/hormonal measures (glucose-6-phosphate dehydrogenase, glycerol-3-phosphate dehydrogenase, insulin, insulin-like growth factor-1, protein, hematocrit, glucose, urea, glucagon, leptin, lipogenesis, and malic enzyme), were also reported.

## Results

### Included Human Studies

**Figure 1:**
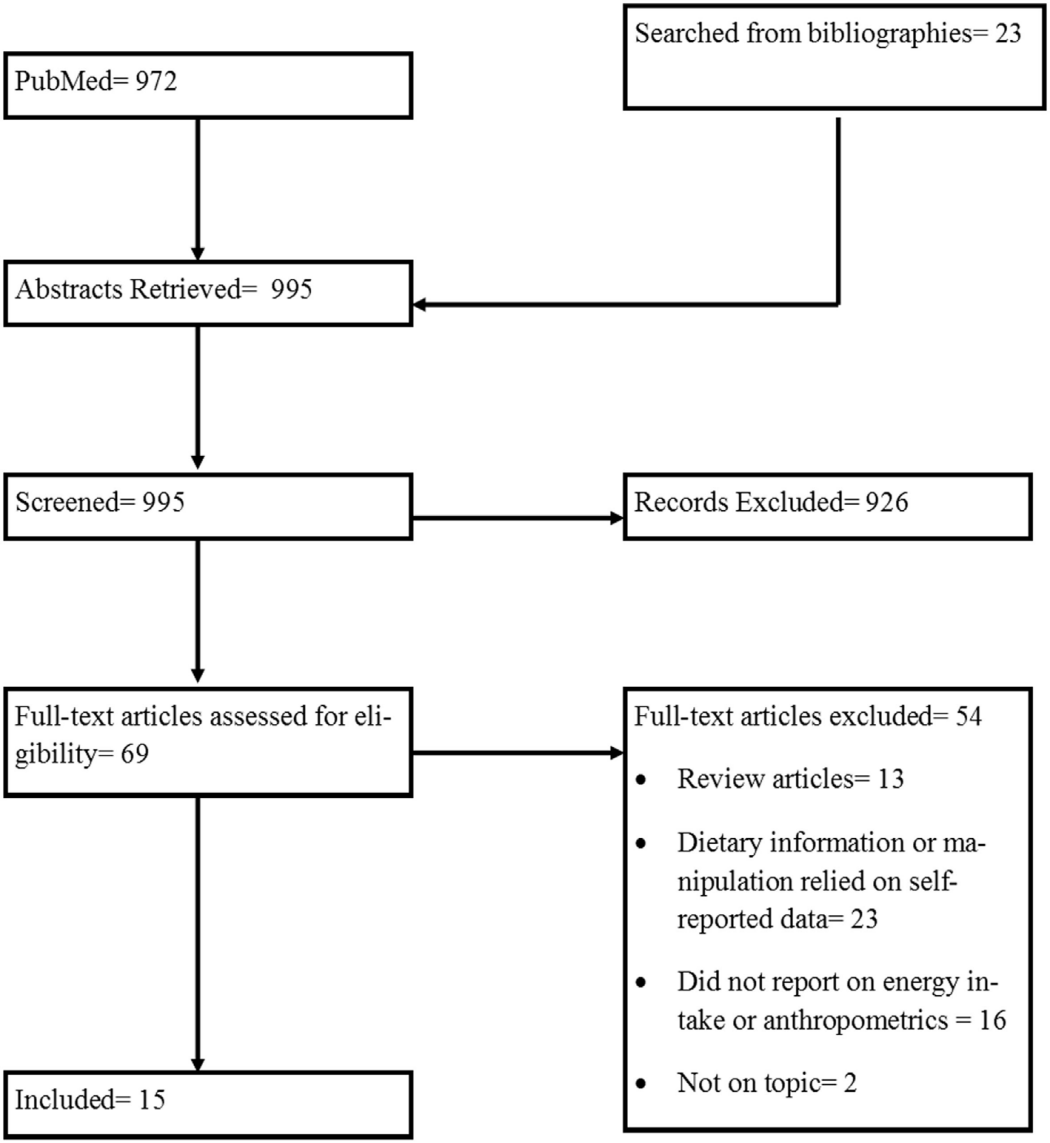
**Diagram of process of selecting included human studies**.

The initial search for human studies yielded 972 records. After removing duplicate records and including additional relevant articles identified through bibliographies of included records, 69 articles were assessed for eligibility and 15 were included in this review. The flow of included studies is outlined in Figure 1. Details of the included human studies are presented in Tables 1 and 2. Table 1 includes 11 studies conducted in laboratory/metabolic ward settings (20–30). Five studies implemented in the field are reported in Table 2 (one study was included in both Tables 1 and 2 as this study had some measures collected in laboratory/metabolic settings in which EF was manipulated, while other measures were collected when EF was manipulated within a field setting) (22, 31–34). All extracted outcomes in the tables are reported as either significant (with direction of significance described), not significant, or no report of significance when EF manipulations were compared. Details of the studies and extracted outcomes are reported below.

**Table 1:** **Eating frequency prescription implemented in laboratory/metabolic ward in human participants**.

Citation	Participants	Study design	EF prescription	Diet prescription	Intervention length and assessments	Measures taken	Results				

							*Ad libitum* meal	Anthropometrics	Energy expenditure	Self-reported appetite regulation	Cardiometabolic/hormonal
Allirot et al. (20)	*Age*: 27.1 ± 1.3 years *BMI*: 22.0 ± 0.3 kg/m^2^ *Gender*: 0% F	RC: *N* = 20	*F1*: breakfast consumed in 1, 20-min bout *F4*: breakfast consumed in 4 equally sized, 10-min bouts every 60 min	674.8 kcal of conventional foods; required to consume all provided foods	4, 240-min laboratory sessions with 7-d between sessions; *ad libitum* lunch meal in 2 sessions at 240 min; EE calculated over 240 min; self-reported appetite regulation measured in 2 sessions, 6× over 240 min; cardio-metabolic/hormonal measured in 2 sessions, all except TG, 17× over 240 min; TG 10× over 240 min	*Ad libitum meal*: objectively measured EI *EE*: DIT measured indirectly using DELTATRAC II calorimeter and QUARK RMR *Self-reported appetite regulation*: hunger, satiety using 70 mm VAS *Cardio-metabolic/hormonal*: AUC for plasma GLP-1, ghrelin, glucose, insulin, and TG	≠		F4 ↓ F1	*Hunger*: F4 ↓ F1 at T240 *Satiety*: F4 ↑ F1 at T240	*GLP-1*: F4 ↓ F1 *Ghrelin*: ≠ *Glucose*: ≠ *Insulin*: F4 ↓ F1 *TG*: ≠
Allirot et al. (23)	*Age*: 28.6 ± 1.5 years *BMI*: 31.9 ± 0.4 kg/m^2^ *Gender*: 0% F	RC: *N* = 17	*F1*: breakfast consumed in 1, 20 min bout *F4*: breakfast consumed in 4 equally sized, 10 min bouts every 60 min	674.8 kcal of conventional foods; required to consume all provided foods	4, Laboratory sessions of varying length with 7-d between sessions; EE measured during basal period (T-30 to 0 min) and over 430 min; self-reported appetite regulation measured in 2 sessions, 9× over 390 min with an *ad libitum* meal at 240 min; cardiometabolic/hormonal measured in 2 sessions, 23× over 430 min with a standardized lunch in which pts were required to eat all foods provided served at T240	*Ad libitum meal*: objectively measured EI *EE*: DIT measured indirectly using DELTATRAC II calorimeter and QUARK RMR *Self-reported appetite regulation*: hunger, satiety using 70 mm VAS *Cardio-metabolic/hormonal*: AUC for plasma GLP-1, ghrelin, glucose, and insulin	≠		NR	*Hunger*: F4 ↓ F1 at T240 *Satiety*: F4 ↑ F1 at T240	*GLP-1*: ≠ *Ghrelin*: ≠ *Glucose*: ≠ *Insulin*: F4 ↓ F1
Antoine et al. (24)	Mean ± SD of pts age and BMI for entire sample NR *Gender*: 100% F	RC: *N* = 10	*Three meal*: 3 meals/day *Six meal*: 6 meals/day	1200 kcal; not specified if required to consume all provided foods	2, 14-d interventions in metabolic ward without washout period; assessments occurred at 1, 15, and 29 d	*Anthropometrics*: BW *Cardio-metabolic/hormonal*: fasting cholesterol, TG		≠			*Cholesterol*: ≠ *TG*: ≠
Bortz et al. (25)	Mean ± SD of pts age and BMI for entire sample NR *Gender*: 100% F	NRC: *N* = 6	*1-Feeding*: 1 meal/day *3 Feeding*: 3, equal-sized meals/day *9-Feeding*: 9, equal-sized meals/day every 2 h	600 kcal/day liquid meals; unspecified if required to consume all provided foods	Intervention in metabolic ward of unspecified length with washout period unspecified; BW and cardiometabolic/hormonal measures every 6 d	*Anthropometrics*: BW *Cardio-metabolic/hormonal*: cholesterol, TG		No difference with significance NR			*Cholesterol*: ≠ *TG*: ≠
Chapelot et al. (26)	Mean ± SD of pts age and BMI for entire sample NR *BMI*: *Snackers*: 21.7 ± 0.4 kg/m^2^ *Meal-eaters*: 21.7 ± 0.4 kg/m^2^ *Gender*: 0% F	NRC: *N* = 12	*Snackers*: regularly ate 4 meals/day and prescribed 3 meals/day *Meal-eaters*: regularly ate 3 meals/day and prescribed 4 meals/day	No prescribed diet	2, 24-h laboratory sessions with 28-d between sessions following prescription; all meals and snack *ad libitum* with breakfast and lunch at standard times and pts required to request afternoon snack and dinner; self-reported appetite regulation measured every 30 min; cardiometabolic/hormonal measured between meals	*Ad libitum meals*: objectively measured EI *Anthropometrics*: BMI *Self-reported appetite regulation*: hunger measured using 100 mm VAS *Cardio-metabolic/hormonal*: AUC for plasma glucose, insulin, leptin, and TAG	≠	≠		NR	*Glucose*: ≠ *Insulin*: ≠ *Leptin*: ≠ *TAGs*: ≠
Dallosso et al. (27)	Mean ± SD of pts age and BMI for entire sample NR *Gender*: 0% F	RC: *N* = 8	*Gorging*: 2 equal-sized meals/day every 8 h *Nibbling*: 6 equal-sized meals/day every 2 h	42 kcal/kg body weight daily for a 65-kg reference man with light activity pattern; unspecified if required to consume all provided foods	2, 14-d interventions in metabolic ward with washout period unspecified; BW and EE measured at 0, 7, 14, 21, and 28 d	*Anthropometrics*: BW *EE*: indirect using whole body indirect open-circuit calorimeter		Gorging ↑ Nibbling at 7 d	≠		
Dougkas et al. (28)	*Age*: 32 ± 9 years *BMI*: 27 ± 2 kg/m^2^ *Gender*: 0% F	RC: *N* = 40	*Snack (3 sessions)*: received a dairy snack (cheese, milk, and yogurt) between breakfast and *ad libitum* lunch *No snack (1 session)*: received water between breakfast and *ad libitum* lunch	Standard breakfast of 348 kcal and in snack sessions 201 kcal of dairy snack; unspecified if required to consume all provided standard breakfast; snack required to be consumed in 5 min	4, 230-min laboratory sessions with 1 week between sessions; *ad libitum* meal occurred at 210 min; self-reported appetite regulation measured 10× over 230 min; cardiometabolic/hormonal measured over 230 min	*Ad libitum meal*: objectively measured EI *Self-reported appetite regulation*: hunger, fullness measured by 100 mm VAS after *ad libitum* meal *Cardio-metabolic/hormonal*: post-prandial plasma cholesterol, ghrelin, glucose, PYY, TAG, and serum insulin	All snacks ↓ water (no snack)			*Hunger*: ≠ *Fullness*: ≠	*Cholesterol*: NR *Ghrelin*: milk and yogurt ↓ water (no snack) *Glucose*: ≠ *PYY*: all snacks ↑ water (no snack) *TAG*: NR *Insulin*: all snacks ↑ water (no snack)
Speechly and Buffenstein (29)	*Age*: 22.9 ± 4.2 years *BMI*: 23.1 ± 2.8 kg/m^2^ *Gender*: 0% F	RC: *N* = 8	*Single*: breakfast in 1 bout *Multi*: breakfast in 5 equal-sized bouts every h	33% EER; required to consume all provided foods	2, 405-min laboratory sessions with unspecified washout period; *ad libitum* meal occurred at 330 min; self-reported appetite regulation measures occurred at 0, 15, 75, 135, 195, 255, and 315 min, and then at 345, 375, and 405 min (15-, 45-, and 75-min after *ad libitum* meal); cardiometabolic/hormonal measures taken the same times as appetite regulation without a measure at 315	*Ad libitum meal*: objectively measured EI *Self-reported appetite regulation*: hunger measured by 100 mm VAS *Cardio-metabolic/hormonal*: plasma glucose and insulin	Single ↑ Multi			≠	*Glucose*: ≠ *Insulin*: Single ↑ Multi at 15 min; Multi ↑ Single at 255 min
Speechly et al. (30)	*Age*: 37.4 ± 18.5 years *BMI*: 40.0 ± 10.9 kg/m^2^ *Gender*: 0% F	RC: *N* = 7	*Single*: breakfast in 1 bout *Multi*: breakfast in 5 equal-sized bouts every h	33% EER; required to consume all provided foods	2, 405-min laboratory sessions with unspecified washout period; *ad libitum* meal occurred at 330 min; self-reported appetite regulation and cardiometabolic/hormonal measures occurred at 0, 15, 75, 135, 195, 255, and 315 min and then at 330, 345, 375, and 405 min (0-, 15-, 45-, and 75-min after *ad libitum* meal)	*Ad libitum meal*: objectively measured EI *Self-reported appetite regulation*: hunger measured by 100 mm VAS *Cardio-metabolic/hormonal*: plasma glucose and insulin	Single ↑ Multi			Single ↑ Multi at 315 min	*Glucose*: ≠ *Insulin*: ≠
Swindells et al. (21)	Mean ± SD of pts age and BMI for entire sample NR *Gender*: 0% F	NRC: *N* = 6	*2 Meals*: 2, equal-sized meals/day *3 Meals*: 3, equal-sized meals/day *9 Meals*: 9, equal-sized meals/day every 2 h	100% of EER; unspecified if required to consume all provided foods	27-d intervention in metabolic ward (6 d of 3 meals, 6 d of 2 meals, 6 d of 3 meals, 6 d of 9 meals, 3 d of 3 meals) with no washout period; BW measures every day	*Anthropometrics*: BW		No difference with significance NR			
Verboeket-van de Venne et al. (22)^a^	Mean ± SD of pts age and BMI of entire sample NR *Gender*: 0% F	RC: *N* = 10	*Gorging*: 2 meals/day (40% energy at 12 p.m. and 60% energy at 6 p.m.) *Nibbling*: 7 meals/day (15% energy at 7:30 a.m., 10% at 10 a.m., 25% energy at 12 p.m., 10% energy at 2 p.m., 5% energy at 4 p.m., 25% energy at 6 p.m.)	Average daily energy requirement based on 7 d food record; instructed to consume all provided foods and asked to return any foods not consumed	2, 1-week interventions (6 d free-living in which food was provided) and 1 d in respiration chamber without washout period	*EE*: DIT measured indirectly in respiration chamber			≠		

*^a^Weight reported in Table 2*.

*Cardiometabolic/hormonal measures are described as what was noted in the original reference. EF, eating frequency; BMI, body mass index; F, female; RC, randomized crossover; *N*, number; min, minute; EE, energy expenditure; TG, triglyceride; EI, energy intake; DIT, dietary-induced thermogenesis; RMR, resting metabolic rate; VAS, visual analog scale; AUC, area under the curve; GLP-1, glucagon-like peptide; ≠, not significant; NR, not reported; SD, standard deviation; BW, body weight; NRC, non-randomized crossover; TAG, triacylglycerol; PYY, peptide YY; EER, estimated energy requirements*.

**Table 2:** **Eating frequency prescription implemented in the field in human participants**.

Citation	Participants	Study design	EF prescription	Diet prescription	Intervention length and assessments	Measures taken	Results				

							Diet	Anthropometrics	Energy expenditure	Self-reported appetite regulation	Cardiometabolic/hormonal
Finkelstein and Fryer (31)	Mean ± SD of pts age and BMI for the entire sample NR *Gender*: 100% F	RCT	*6 meals*: *N* = 4; 6 meals/day every 2.5 h *3 meals*: *N* = 4; 3 meals/day every 5 h and 1 evening snack/day	Low-kcal (1400 kcal), low-fat (40 g fat) with all foods provided	60-d intervention; BW measured every 7th d; all cardiometabolic/hormonal measures except glucose measured at fasting on d 0 and 60; fasting glucose measured every 7th d	*Anthropometrics*: BW *Cardio-metabolic/hormonal*: glucose, total serum cholesterol, lipids		≠			*Glucose*: ≠ *Cholesterol*: significance NR *Lipids*: ≠
Iwao et al. (33)	Mean ± SD of pts age and BMI for entire sample NR; gender of entire sample not reported *Age*: *2M*: 20.3 ± 0.3 years *6M*: 19.7 ± 0.5 years	RCT	*2M*: *N* = 6; 2, equal-sized meals/day every 12 h *6M*: *N* = 6; 6, equal-sized meals/day every 2 h	Low-kcal (1200 kcal), low-fat (19.8% energy from fat) with all food provided as a liquid supplement; unspecified if required to consume all provided foods	14-d intervention; assessments occurring at 0 and 14 d, cardiometabolic/hormonal measures measured at fasting	*Anthropometrics*: BW *EE*: measured indirectly using DELTA-TRAC calorimeter *Cardio-metabolic/hormonal*: plasma free fatty acids		≠	≠		≠
Murphy et al. (32)	*Age*: 22 ± 0.9 years *BMI*: 23.6 kg/m^2^ (SD NR) *Gender*: 100% F	RC: *N* = 11	*Gorging*: 2 meals/day every 7.5 h *Nibbling*: 12 meals/day every h	2000 kcal; unspecified if required to consume all provided foods	2, 2-week interventions with 3 weeks between interventions; assessments occurred at 0, 2, and 7 weeks with cholesterol, HDL, and LDL cholesterol measures taken at fasting and all other cardiometabolic/hormonal measures taken over 8 h	*Diet*: EF and EI measured using 3, 24-h recalls *Cardio-metabolic/hormonal*: plasma cholesterol, GIP, GLP-1, glucose, HDL, insulin, LDL, TAG	*EF*: Nibbling ↑ Gorging (significance NR) *EI*: ≠				*Cholesterol*: ≠ *GIP*: ≠ *GLP-1*: ≠ *Glucose*: ≠ *HDL*: Nibbling ↓ Gorging *Insulin*: ≠ *LDL*: ≠ *TAG*: ≠
Stote et al. (34)	*Age*:45.0 ± 0.7 yr *BMI*: 23.4 ± 0.5 kg/m^2^ *Gender*: 66.6% F	RC: *N* = 15	*One Meal*: 1 meal/day in 4 h period in evening *Three Meal*: 3 meals/day with meal spacing guidelines unspecified	BEE x 1.3-1.5; required to consume all provided foods	2, 8-week interventions with 11 weeks between interventions; BW measured daily before evening meal; self-reported appetite regulation measures taken daily before evening meal; cardiometabolic/hormonal measures taken at fasting at 0, 4, and 8 weeks of each intervention	*Anthropometrics*: BW *Self-reported appetite regulation*: hunger, fullness self-report using 100 mm VAS *Cardio-metabolic/hormonal*: plasma cholesterol, glucose, HDL, LDL TAG		One meal ↓ Three meal		*Hunger*: One meal ↑ Three meal *Fullness*: One meal ↓ Three meal	*Cholesterol*: One meal ↑ Three meal *Glucose*: ≠ *HDL*: One meal ↑ Three meal *LDL*: One meal ↑ Three meal *TAG*: ≠
Verboeket-van de Venne et al. (22)	Mean ± SD of pts age and BMI for entire sample NR *Gender*: 0% F	RC: *N* = 10	*Gorging*: 2 meals/day (40% energy at 12 p.m. and 60% energy at 6 p.m.) *Nibbling*: 7 meals/day (15% energy at 7:30 a.m., 10% at 10 a.m., 25% energy at 12 p.m., 10% energy at 2 p.m., 5% energy at 4 p.m., 25% energy at 6 p.m.)	Based on average daily energy requirement based on a 7-d food record; instructed to consume all provided foods and asked to return any foods not consumed	2, 1-week interventions (6-d free-living in which food was provided and 1 d in respiration chamber) without washout period; BW measured at beginning and end of each 1-week period; average daily metabolic rate measured over 6-d in free-living conditions	*Anthropometrics*: BW *EE*: doubly labeled water (mean calculated for whole week)		≠	≠		

*Cardiometabolic/hormonal measures are described as what was noted in the original reference. EF, eating frequency; SD, standard deviation; BMI, body mass index; NR, not reported; F, female; RCT, randomized controlled trial; *N*, number; BW, body weight; ≠, not significant; RC, randomized crossover; HDL, high-density lipoprotein; LDL, low-density lipoprotein; EI, energy intake; GIP, gastric inhibitory polypeptide; GLP-1, glucagon-like polypeptide-1; TAG, triacylglycerol; BEE, basal energy expenditure; VAS, visual analog scale; EE, energy expenditure*.

#### Studies Conducted in Laboratory/Metabolic Ward Settings

Out of the 11 studies conducted in laboratory/metabolic ward settings, 4 manipulated EF within one eating occasion (breakfast), while the remaining 7 studies manipulated EF occurring over a longer period (20–30). Eight studies used a randomized crossover design (20, 22–24, 27–30). EF manipulations varied greatly between the 11 studies, ranging from 1 to 9 eating occasions per experimental session or day. Length of manipulation of EF also varied, ranging from 230 min to 14 days. Sample size per condition ranged from 6 to 40 participants. Six studies included an *ad libitum* meal measure taken after the EF manipulation, which ranged from 230 min to 24 h (*ad libitum* meals were measured toward the end of the manipulations), and results for this measure were mixed, with three studies showing significantly lower *ad libitum* intake in conditions of higher EF, and three studies showing no significant difference in intake between EF conditions (20, 23, 26, 28–30). Of the five studies measuring anthropometrics, two found no significant differences between EF conditions, two described no differences between EF conditions but significance was not reported, and one found a significantly lower outcome in the higher EF condition (21, 24–27). Energy expenditure was measured in four studies, while no study reported that increased EF enhanced energy expenditure, one study reported that a higher EF produced a significantly lower dietary-induced thermogenesis, and two studies reported no significant effect of EF on energy expenditure (20, 22, 23, 27). Measures of self-reported appetite, collected in various ways and at various time points during the EF manipulation, were reported in six studies with mixed outcomes, with three studies reporting significantly less self-reported hunger in the conditions with higher EF, two reporting no significant difference between conditions, and one not describing outcomes (20, 23, 26, 28–30). Eight studies examined cardiometabolic/hormonal measures, with six studies finding no significant effect of EF in the majority of the measures taken (20, 23–26, 28–30). In these eight studies, glucose was measured in six of the investigations, with all six studies finding no significant effect of EF, and insulin was measured in the same six studies, with outcomes being mixed (two studies reported significantly lower insulin in the higher EF conditions; one study reporting significantly lower insulin in the higher EF condition when the measure was taken 15 min into the session, but significantly higher insulin 255 min into the session; one study reporting significantly higher insulin in the higher EF condition; and two studies reporting no significant difference between conditions) (20, 23, 26, 28–30).

#### Studies Conducted in Field Settings

Out of the five studies conducted in field settings, three used a randomized crossover design (22, 31–34). For the 5 studies, the number of eating occasions in the EF manipulations varied greatly, ranging from 1 to 12 eating occasions per day, intervention length ranged from 1 to 8.5 weeks, and sample size per condition ranged from 4 to 15 participants (22, 31–34). The one study reporting on energy intake found no significant difference in energy intake between EF conditions (32). Four studies reported on weight, with three finding no significant difference between EF conditions, and one reporting a significantly greater weight in the higher EF condition (22, 31, 33, 34). One study reported on energy expenditure and found no significant difference between conditions (22), and one study reported on self-reported appetite and found that hunger was significantly lower and fullness was significantly greater in the higher EF condition (34). For the four studies reporting on cardiometabolic/hormonal measures, cholesterol outcomes were mixed (one did not report significance, one reported no significant effect of EF, and one reported that the higher EF condition was significantly lower than the lower EF condition), but all studies found no significant effect of EF on glucose (31–34).

### Included Animal Studies

**Figure 2:**
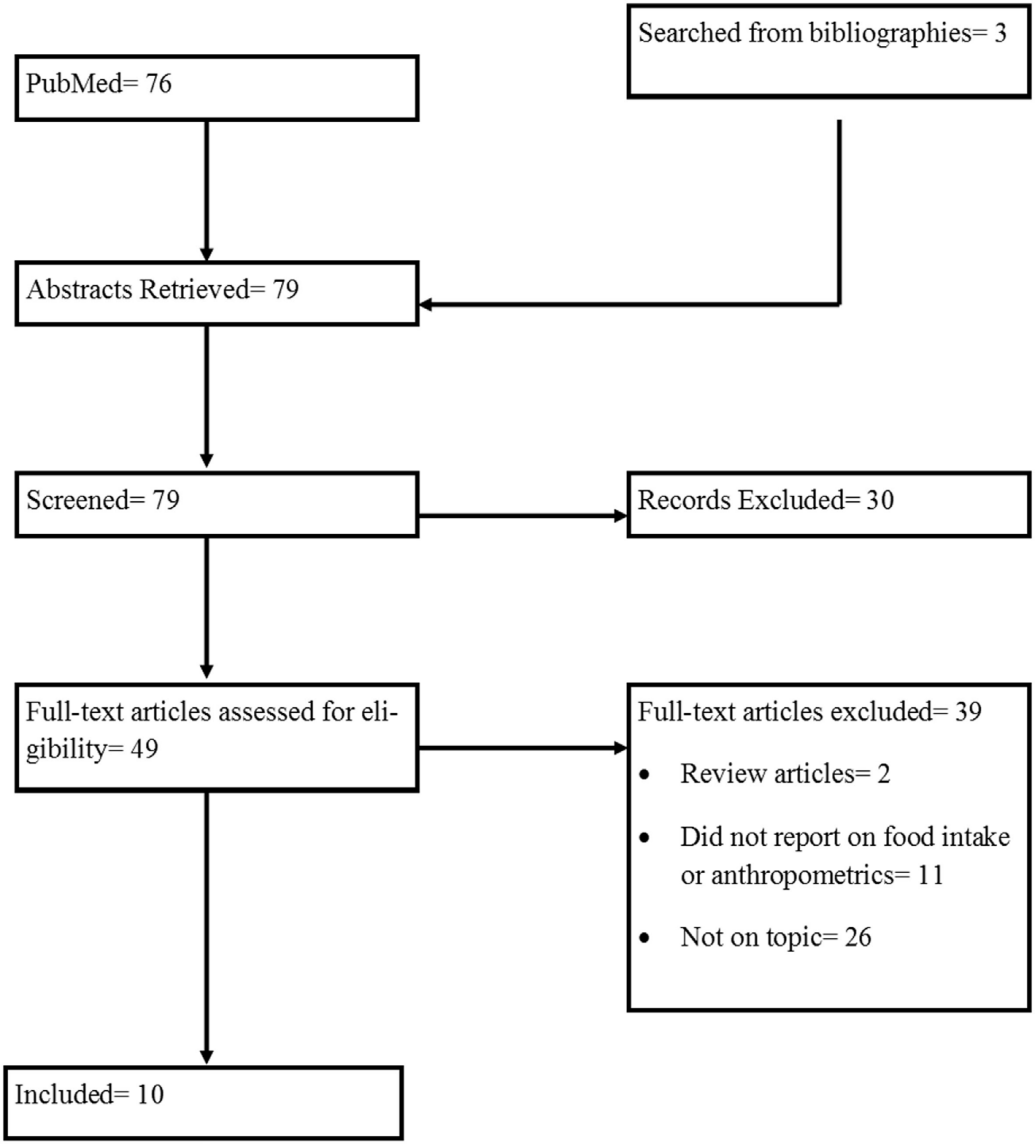
**Diagram of process of selecting included animal studies**.

The initial search for animal studies yielded 76 results. After removing duplicate records and including additional relevant articles identified through the bibliographies of included records, three additional articles were assessed for eligibility. Of the 79 articles chosen for the screening process, 10 were selected to be included in this review. The flow of included studies is outlined in Figure 2. The details of the animal studies included are outlined in Tables 3 and 4.

**Table 3:** **Experimental period <1 month in animal studies**.

Citation	Animal model	Comparison groups	EF manipulation	Diets	Testing duration	Measures taken	Results		

							Food intake	Anthropometrics	Cardiometabolic/hormonal
Anderson et al. (35)	Rat pups (no specific model system noted)	*LFF*: *N* = 56; 4×/d *HFF*: *N* = 14; 24×/d	Gastrostomy coupled with HFF vs. LFF	Gastrostomy tubes inserted 24 h of age; standard formula provided with daily amount = 0.5 kcal/g body weight	5–7 d	*Anthropometrics*: BW gain, organ weights *Cardiometabolic/hormonal*: G6PDH and GPDH		*BW gain*: HFF ↓ LFF *Organ weights*: HFF ↓ LFF (small intestine, liver, stomach) HFF ↑ LFF (spleen)	*G6PDH*: HFF ↓ LFF *GPDH*: HFF ↓ LFF
Atalayer and Rowland (36)	Albino mice	*4*×/d: *N* = 5 *8*×*/*d: *N* = 5 *16*×/d: *N* = 5	Feeding opportunities available during 12 h dark period and 1st 4 h of light period: 4×/d = access to food for 40 min at beginning of every 4th h; 8×/d = access to food for 20 min at beginning of every 2nd h; 16×/d = access to food for 10 min at beginning of every h; all groups had 160 min/d access to food; size of feeding opportunity depend on food cost (an additional manipulation)	20 mg Purina chow pellets (10.4% kcal from fat + 24.1% kcal from protein)	3–4 d	*Food intake*: total intake *Anthropometrics*: BW	≠	≠	
DeVries et al. (37)	Holstein cows	Experiment 1: *1*×*/*d: *N* = 12; *2*×*/*d: *N* = 12 Experiment 2: *2*×*/*d: *N* = 12; *4*×/d: *N* = 12	1×/d at 5:30 a.m.; 2×/d at 5:30 a.m. and 3:15 p.m.; 4×/d at 5:30 a.m., 11:00 a.m., 3:15 p.m., and 10:30 p.m. Amounts offered per feeding or across the d NR	Experiment 1: total mixed ration 1 (51.2% concentrate and 48.8% forage) Experiment 2: total mixed ration 2 (52.2% concentrate and 47.8% forage)	3 d adjustment period followed by 7 d observation period	*Food intake*: total intake	≠ (In both exp. 1 and 2)		
Nussbaum et al. (38)	Simmental-red Holstein calves, Braunvieh-Brown Swiss calves, and Holstein Friesian calves	*LFF*: *N* = 7; 2×/d *HFF*: *N* = 7; ≥6×/d	LFF at 8:00 a.m. and 5:00 p.m. by bucket feeding; HFF ranged from 6 to 14×/d by automated computer feeding Total daily amount provided identical	Colostrum (d 1–3) followed by mature milk powder (d 4–14) and finally mature milk (d 15–28); bucket feeding of colostrum and milk powder contained water in concentration of 4 g/100 g and 5 g/100 g, respectively; automated feeding ranged from 0.5 to 1.5 L per portion	28 d	*Anthropometrics*: BW *Cardiometabolic/hormonal*: plasma hematocrit, IGF-1, insulin, protein		≠	*Hematocrit*: ≠ *IGF-1*: HFF ↑ LFF *Insulin*: HFF ↑ LFF *Protein*: HFF ↓ LFF
Vicari et al. (39)	Holstein-Friesian Calves	*1*×/d *2*×*/*d *4*×*/*d *N* = 15; NR how many were in each EF in period 2	1×/d at 12:00 p.m.; 2×/d at 12:00 p.m. and 12:00 a.m.; 4×/d at 6:00 a.m., 12:00 p.m.; 6:00 p.m., and 12:00 a.m. Amount fed was based on MEm Period 1: experimental diet fed at low feeding level (1.5× MEm, 36.6 g/kg/day) in all 3 groups Period 2: experimental diet fed at low feeding level in 1×/d group and high feeding level (2.5× MEm, 61.1 g/kg/day) levels in 2×/d and 4×/d groups	Experimental milk replacer diet	14 d for period 1; 28 d washout period; 14 d for period 2	*Food intake*: total intake *Anthropometrics*: BW *Cardiometabolic/hormonal*: plasma glucagon, glucose, IGF-1, insulin, leptin, urea	≠	≠	*Glucagon*: 1×/d↑ 2×/d ↑ 4×/d *Glucose*: 2×/d ↑ 4×/d *IGF-1*: ≠ *Insulin*: 2×/d ↑ 4×/d *Leptin*: ≠ *Urea*: ≠

*Cardiometabolic/hormonal measures are described as what was noted in the original reference. EF, eating frequency; LFF, low feeding frequency; *N*, number; d, days; HFF, high feeding frequency; BW, body weight; G6PDH, glucose-6-phosphate dehydrogenase; GPDH, glycerol-3-phosphate dehydrogenase; min, minute; ≠, not significant; NR, not reported; IGF-1, insulin-like growth factor-1; MEm, metabolizable energy requirements for maintenance*.

**Table 4:** **Experimental period >1 month in animal studies**.

Citation	Animal model	Comparison groups	EF manipulation	Diets	Testing duration	Measures taken	Results		

							Food intake	Anthropometrics	Cardiometabolic/hormonal
Mantysaari et al. (40)	Finnish Ayrshire Cows	*LFF*: 1×/d *HFF*: 5×/d *N* = 40; NR how many were in each EF	Fed 1×/d vs. 5×/d Amounts offered per feeding or across the d NR	Diet: total mixed ration (grass silage and concentrate mix); concentrate mix contained 60.6% barley, 27% rapeseed meal, 10% molasses sugar beet pulp, and 2.4% vitamin and mineral mix	From calving to 28 weeks lactation	*Food intake*: total intake *Anthropometrics*: BW	HFF ↓ LFF	≠	
Muiruri and Leveille (42)	Sprague-Dawley rats	*AdL*: *N* = 25 *1*×*/*d: *N* = 20; 1, 2 h access *2*×*/*d: *N* = 5; 2, 1 h access	*First 3 weeks (adaptation period)*: groups 1, 3, and 4: fed 1×/d; 8:00 a.m. to 10:00 a.m.; group 2: fed AdL *Second 3 weeks*: group 1: fed 1×/d at 8:00 a.m. to 10:00 a.m.; group 3: fed 2×/d at 8:00 a.m. to 9:00 a.m. and 4:00 p.m. to 5:00 p.m.; groups 2 and 4: fed AdL Amounts offered per feeding or across the d NR	Purified diet (70% glucose, 19% casein, and 12% fat)	6 weeks (3 weeks adaptation; 3 weeks experiment)	*Food intake*: total intake *Anthropometrics*: BW gain, body fat% gain *Cardiometabolic/hormonal*: adipose tissue lipogenesis, G6PDH, 6PGD, malic enzyme	Group 4 ↑ Group 1, 2, 3 Group 2, 3 ↑ Group 1	*BW gain*: Group 4 ↑ Group 1, 2, 3; Group 3 ↑; Group 1, 2 *Body fat% gain*: Group 3 ↑ Group 1, 2, 4	*Lipogenesis*: Group 1 ↑ Group 2, 3, 4; Group 3, 4 ↑ Group 2 *G6PDH*: Group 1, 3 ↑ Group 2, 4; Group 4 ↑ Group 2 *6PGD*: Group 1, 3 ↑ Group 2, 4; Group 4 ↑ Group 2 *Malic enzyme*: Group 1 ↑ Group 2, 3, 4; Group 3 ↑ Group 2, 4; Group 4 ↑ Group 2
Robles et al. (43)	Holstein Heifers	*1*×*/*d *2*×*/*d *3*×*/*d *4*×*/*d *N* = 4; NR how many were in each EF	1×/d at 8:00 a.m.; 2×/d at 8:00 a.m. and 8:00 p.m.; 3×/d at 8:00 a.m., 2:00 p.m., and 8:00 p.m.; 4×/d at 8:00 a.m., 12:00 p.m., 4:00 p.m., and 8:00 p.m. Total daily amount provided identical	Concentrate diet and barley straw	4, 2-week periods	*Food intake*: total intake	≠		
Steelman et al. (44)	Quarter Horse Yearlings	*2*×*/*d: *N* = 3 *3*×*/*d: *N* = 3 *4*×*/*d: *N* = 3	2×/d at 7:00 a.m. and 7:00 p.m.; 3×/d at 7:00 a.m., 3:00 p.m., and 11:00 p.m.; 4×/d at 1:00 a.m., 7:00 a.m., 1:00 p.m., and 7:00 p.m. Total food offered was constant at 2.5% BW/day Amounts offered per feeding not reported in detail, but all horses were reported to have equal access to hay	Concentrate diet (pellets) and Bermuda grass hay	33 d	*Anthropometrics*: BW *Cardiometabolic/hormonal*: plasma glucose (0.5, 1.5, 2.5, 3.5, 4.5, and 5.5 h after feeding on d 11), serum leptin (starting at 6:00 p.m. and then every 2 h in the last 24 h)		≠	*Glucose*: 2×/d ↑ 3×/d, 4×/d at 0.5 and 2 h after feeding *Leptin*: 2×/d ↑ 4×/d ↑ 3×/d at 10:00 p.m.
Wu et al. (41)	Rare Minnow	*1*×*/*d *2*×*/*d *3*×*/*d 80 juvenile fish allocated in triplicate groups for a total of 13 groups	Factorial experiment of temperature × feeding frequency Temperatures: uncontrolled ambient temperature (16.3–19.2°C), T20 (20°C), T24 (24°C), T28 (28°C) Feeding was conducted to satiation	NR	8 weeks	*Anthropometrics*: BW gain		2×/d > 3×/d > 1×/d at ambient temp 3×/d > 2×/d > 1×/d at 20°C 2×/d > 3×/d > 1×/d at 24°C 3×/d > 2×/d > 1×/d at 28°C

*Cardiometabolic/hormonal measures are described as what was noted in the original reference. EF, eating frequency; LFF, low feeding frequency; d, days; HFF, high feeding frequency; NR, not reported; BW, body weight; ≠, not significant; AdL, *ad libitum*; G6PDH, glucose-6-phosphate dehydrogenase; 6PGD, 6-phosphogluconate dehydrogenase*.

Table 3 describes five EF studies that had a testing duration of <1 month (35–39). Table 4 describes five EF studies that had a testing duration of >1 month (40–44). All extracted outcomes in the tables are reported as either significant (with direction of significance described), not significant, or no report of significance when EF manipulations were compared. Details of the studies and extracted outcomes are reported below.

#### Experimental Period <1 month

The five studies with an experimental period of <1 month included an assortment of animal models, such as rat pups, mice, calves, and cows, with 5–56 animals per condition (35–39). For the five studies, the number of feeding occasions in the EF manipulations varied greatly, ranging from 1 to 24 feeding occasions per day, and the experimental period ranged from 3 to 28 days (35–39). Of the three studies reporting on intake, all found no significant difference in consumption between EF conditions (36, 37, 39). Of the four studies reporting on body weight, three found no significant difference between EF conditions, and one found significantly less body weight gain in the higher EF condition (35, 36, 38, 39). Cardiometabolic/hormonal measures taken varied between studies, but insulin and insulin-like growth factor-1 were measured in two studies, with inconsistent outcomes found (one study found significantly higher insulin in the higher EF condition, while the other study found significantly lower insulin in the higher EF condition; one study found significantly higher insulin-like growth factor-1 in the higher EF condition, while the other study found no difference among the EF conditions) (38, 39).

#### Experimental Period >1 month

The five studies with an experimental period of >1 month included cow, rat, horse, and rare minnow models, with 3–25 animals per condition (40–44). For the five studies, the number of feeding occasions in the EF manipulations varied from 1 to 5 occasions, and experimental length ranged from 33 days to 28 weeks (40, 42–44). Of the three studies reporting on intake, results were mixed, with one study finding significantly less intake in the higher EF condition, one study finding significantly greater intake in the higher EF condition, and one finding no significant difference in intake in the EF conditions (40, 42, 43). Two of the four studies reporting on body weight found no significant effect of the EF manipulation, one study with a factorial design did not find a main effect of EF on body weight gain, and one study found greater weight gain with higher EF (40–42, 44). Only two studies collected cardiometabolic/hormonal measures, and the measures collected were not the same (42, 44).

## Discussion

The purpose of this systematic review was to provide a comprehensive review of experimental research conducted in both humans and animals in the areas of greater EF, food intake, and body weight. Twenty-five studies, using varying study designs, EF manipulations, and lengths of experimentation, were identified and included in the review (20–44). As a whole, the reviewed experimental studies provide little support that increasing EF influences intake or body weight. Out of the 13 studies that reported on a measure of consumption, 8 (61.5%) found no significant effect of EF (20, 23, 26, 28–30, 32, 36, 37, 39, 40, 42, 43). Seventeen studies reported on body weight or BMI, with 11 studies (64.7%) finding no significant effect of EF (21, 22, 24–27, 31, 33–36, 38–42, 44).

When potential mechanisms were examined, four studies reported on the effect of greater EF on energy expenditure (one study reported on two different measures of energy expenditure), with two studies (50.0%) finding no influence of EF, one study reporting reduced energy expenditure with greater EF, and one study not reporting significance (20, 22, 23, 27). Self-reported appetite measures were collected in various ways across the investigations and outcomes were mixed, but slightly leaning toward a greater EF reducing hunger (57.1%) (20, 23, 26, 28–30, 34). The cardiometabolic/hormonal measures also greatly varied, both in terms of type and methodology regarding when the measures were taken. Of the cardiometabolic/hormonal measures, glucose and insulin were the most commonly taken measures (11 and 9 studies, respectively) (20, 23, 26, 28–32, 34, 38, 39, 44). Glucose measures consistently found no effect of greater EF (81.8% of studies) and insulin measures were mixed (20, 23, 26, 28–32, 34, 38, 39, 44).

The hypothesis that increased EF may influence energy intake and/or anthropometrics continues to be sustained in the literature. For example, evidence cited in previous reviews on EF have suggested that greater EF may not have a strong impact on energy intake and/or anthropometrics, yet these reviews have still concluded that there may be an effect of EF on energy intake or anthropometrics (9, 12–15). These reviews have included observational research, or only specific types of experimental research in humans (i.e., controlled feeding studies, studies prescribing a hypocaloric diet), in which the implementation of the increased EF prescription may not have been reported. This is the first review that included only experimental research, in both humans and animals, in the area of greater EF on energy intake and/or anthropometrics. The experimental research included in this review needed to implement the EF manipulation in a controlled manner (i.e., implemented in a research setting, or with food packaged and provided to participants), providing a high degree of internal validity for the EF manipulation. The outcomes of this review show that more than half of studies found no significant difference in energy intake or anthropometrics in differing EF conditions.

As the initial research which suggested that greater EF may influence intake and anthropometrics was observational, and issues about accuracy of self-reported dietary intake have been suggested as a factor in producing results that indicate that greater EF is related to a lower weight status (13), the finding that more than half of the experimental research in which EF and energy intake are objectively measured, did not show a significant reduction in intake or anthropometrics suggests that the relationship found between greater EF and weight status in observational studies may be a consequence of a self-reported dietary artifact. As accuracy of self-reported dietary intake has been shown to be related to weight status (12, 45–48), observational studies examining relationships between dietary variables and weight status should address issues of under-reporting in analysis (i.e., remove under-reporters from the analyses) to help identify variables that may be truly related to weight status.

As this review did not include animal models of diseased states or human participants with health conditions other than overweight or obesity, greater EF may have an influence on energy intake, anthropometrics, and/or cardiometabolic/hormonal outcomes in human or animal models demonstrating impaired glucose or lipid metabolism (i.e., diabetes and cardiovascular disease). Additionally, it has been suggested that physical activity may be an important variable in the relationship between greater EF and energy intake and anthropometrics (49), and the investigations included in this review were not specifically designed to examine the role of physical activity in the relationship. Thus, conclusions about the relationship between greater EF, physical activity, energy intake, and anthropometrics cannot be determined. Furthermore, greater EF may influence energy intake and anthropometrics during weight loss maintenance differently than during weight maintenance or weight loss, or in healthy weight vs. overweight or obese states. As the focus of this review was on examining the influence of increased EF, search terms related to reduced EF (i.e., intermittent fasting, eating occasion omission) were not included in this review. Moreover, as the longest time frame of implementation of the EF manipulations was 8 weeks in humans and 28 weeks in an animal model, studies with an increased EF manipulation over longer time frames may find differences in intake and weight outcomes than what is reported in this review. However, longer time frames can be challenging to implement when studies are designed to contain a high degree of internal validity (i.e., where weighed and measured food is provided to participants or animals so that issues related to self-reported dietary data can be avoided). Future research should examine if any of these variables (i.e., disease states, physical activity, energy balance and weight status, long-term implementation of increased EF) influence the relationship between increased EF and intake and/or anthropometrics.

There are several limitations to this systematic review. Within the studies, there is great diversity of the EF manipulations, the types of measures collected, and when during the EF manipulation the measures were collected. This makes it challenging to pool effect sizes across the investigations. Additionally, the animal studies used several animal models, and the models may have differing “typical” eating patterns (i.e., animals that typically graze vs. animals that do not), producing a potential differential response to EF manipulations. These differing models again make it challenging to pool outcomes. Finally, while many studies used experimental designs that capitalized on using within-subject factors, the sample sizes in the investigations as a whole were small, which could indicate that the studies may be underpowered to detect differences in outcomes between EF conditions. These limitations reduce ability to draw firm conclusions regarding the effect of EF on energy intake and anthropometrics.

In summary, the human and animal experimental studies included in this review suggest that greater EF may not necessarily influence energy intake or anthropometrics. This indicates that contrary to what is commonly proposed in the lay literature, eating more frequently during the day (i.e., “grazing”) may not assist with reducing energy intake or improving weight status.

## Author Contributions

HR and GC developed study premise. HR, MG, and GC reviewed all identified abstracts, and HR and MG reviewed all identified articles. HR, MG, SP, and GC abstracted data from articles. HR, MG, and SP developed initial draft of paper, and all authors contributed to and approved the final version of the manuscript.

## Conflict of Interest Statement

The authors declare that the research was conducted in the absence of any commercial or financial relationships that could be construed as a potential conflict of interest.

## Abbreviations

AUC, area under the curve; BMI, body mass index; EF, eating frequency.
